# Diagnostic moléculaire d'helicobacter pylori par PCR chez les patients en consultation gastroentérologique au Centre Médical Saint Camille de Ouagadougou

**DOI:** 10.11604/pamj.2015.21.123.6001

**Published:** 2015-06-15

**Authors:** Karidia Werme, Cyrille Bisseye, Issiaka Ouedraogo, Albert Théophane Yonli, Djénèba Ouermi, Florencia Djigma, Rémy Moret, Charlemagne Gnoula, Jean-Baptiste Nikiema, Jacques Simpore

**Affiliations:** 1 Centre de Recherche Biomoléculaire Pietro Annigoni (CERBA)/LABIOGENE, Université de Ouagadougou, Burkina Faso; 2 Centre Médical Saint Camille (CMSC) Ouagadougou, Burkina Faso; 3 Laboratoire de Biologie Moléculaire et Cellulaire (LABMC), Equipe Microbiologie/Immunologie, Université des Sciences et Techniques de Masuku (USTM), Franceville, Gabon

**Keywords:** Helicobacter pylori, gastrite, prévalence, PCR, Immunocomb, Burkina Faso, Helicobacter pylori, gastritis, prevalence, PCR, Immunocomb, Burkina Faso

## Abstract

**Introduction:**

L'infection par Helicobacter pylori constitue un problème de santé publique notamment dans les pays en développement. Elle entraine une gastrite pouvant évoluer vers des formes sévères d'ulcération et de transformation maligne. La présenté étude avait pour objectif de diagnostiquer H. pylori par des techniques sérologique et moléculaire au Burkina Faso.

**Méthodes:**

L’étude prospective a été conduite de mars à juin 2012 sur 70 patients venus en consultation dans le service de gastroentérologie au Centre Médical Saint Camille. Le diagnostic de H. pylori a été réalisé par le test ELISA Immunocomb (ORGENICS Ltd, Yavne, Israël) et la PCR sur des biopsies gastriques prélevées sur les patients.

**Résultats:**

Les pathologies gastroduodénales étaient plus fréquentes chez les patients de plus de 45 ans. Les prévalences de H. pylori étaient respectivement de 88,57% et de 91,43% par sérologie Immunocomb et par PCR. La différence entre les deux techniques n’était pas significative (P = 0,573). La performance de la PCR a été comparée à celle de la technique Immunocomb. Les résultats montrent une sensibilité et une spécificité de 92,2% et 50,0% pour la technique Immunocomb.

**Conclusion:**

Le diagnostic de H. pylori par PCR est plus spécifique que le test sérologique Immunocomb et devrait être introduit dans le diagnostic de routine de cette bactérie pathogène au Burkina Faso.

## Introduction

Helicobacter pylori est une bactérie spiralée micro aérophile, Gram négatif, colonisant la muqueuse gastrique des humains avec un mode de transmission interhumain principalement oral-oral. La colonisation par H. pylori commence dans l'enfance, alors que les manifestations cliniques de cette infection ne se développent habituellement qu’à l’âge adulte. La source d'infection la plus probable est la famille proche et/ou des contacts rapprochés mais le mode exact de transmission et les facteurs de susceptibilité à l'infection restent incomplètement connus [1–3]. L'infection par H. pylori est très souvent asymptomatique et touche plus de 50% de la population mondiale [4]. Le taux de prévalence de H. pylori varie selon les pays. En effet, des prévalences de 69% et de 96,1% ont été rapportés respectivement en Cote d'Ivoire et au Burkina Faso [5, 6]. H. pylori est la principale cause des gastrites [7], des ulcères gastroduodénaux [8] et de deux types de néoplasies gastriques: l'adénocarcinome et le lymphome gastrique du tissu lymphoïde associé aux muqueuses (MALT) [9].

Le diagnostic de l'infection repose sur l'examen des biopsies gastriques, culture spécifique, test rapide à l'uréase, PCR ou des méthodes de prélèvement non invasif (test respiratoire à l'urée, sérologie, recherche d'antigène dans les selles). Parmi ces méthodes non-invasives, la sérologie est le test plus utilisé en routine dans le diagnostic de H. pylori au Burkina Faso. Cependant, cette méthode de diagnostic est incapable de différencier une infection active d'une infection guérie par H. pylori à cause de la persistance des anticorps longtemps après la guérison [10]. Des études antérieures ont montré que la sensibilité et la spécificité des tests sérologiques pour la détection de H. pylori variaient entre 80% et 90% [11]. Cette faible sensibilité et spécificité des tests sérologiques pourrait être responsable d'une sous-estimation des infections à H. pylori. Une technique diagnostic devenue incontournable dans la recherche des agents infectieux est la réaction en chaine par polymérase (PCR). Il a été montré que la PCR était une méthode légèrement supérieure aux autres méthodes diagnostics dans la détection de H. pylori et dans l’évaluation de l'efficacité de son traitement [12, 13]. La PCR s'impose de plus en plus comme la méthode de référence dans le diagnostic de H. pylori. En effet, une de ces variantes (PCR nichée) a montré une sensibilité et une spécificité de 100% dans le diagnostic de cette bactérie [14]. En dépit de sa haute sensibilité et spécificité, le diagnostic moléculaire par PCR est peu utilisé dans la détection de H. pylori au Burkina Faso. La présente étude avait pour objectif d’évaluer l'efficacité des techniques sérologique et moléculaire dans le diagnostic de H. pylori chez les patients en consultation gastroentérologique au Centre Médical Saint Camille de Ouagadougou.

## Méthodes

**Recrutement des patients:** le but et la nature de l’étude ont été expliqués à l'ensemble des patients en consultation de gastroentérologie. Seuls les 70 patients ayant consenti librement à participer à l’étude ont été recrutés et leurs données sociodémographiques et cliniques ont été répertoriés dans une fiche d'enquête dûment remplie. Tous les patients ayant refusé de participer à l’étude n'ont pas été retenus. L’étude prospective s'est déroulée sur une période de 4 mois (mars-juin 2012). La majorité des patients provenaient de Ouagadougou et de ses environs.

**Diagnostic de H. pylori:** sur chaque patient, deux types de prélèvements ont été réalisés: une biopsie gastrique pour le diagnostic par PCR et un prélèvement sanguin pour la sérologie. Les fibroscopies ont été réalisées par un fibroscope de type Olympus GIF 100 Prémédication/Xylo gel. Les prélèvements sanguins ont été effectués dans des tubes imprégnés d'EDTA. Les plasmas ont été séparés centrifugation à 2000 rpm pendant 10 minutes et conservés à -20°C dans des cryotubes. Les anticorps anti-Helicobacter pylori ont été recherchés dans les plasmas par le test sérologique ELISA Immunocomb II Helicobacter pylori IgG (ORGENICS Ltd, Yavne, Israël) en suivant les instructions du fabricant. L'ADN à été isolé des biopsies à l'aide du Kit DNA SorbB (Sacace biotechnologie, como, Italie) selon les instructions du fabricant. La PCR a ensuite été effectuée avec un Thermocycleur Applied Biosystems 9700, dans un volume réactionnel de 20µl avec le kit commercial Helicobacter pylori 520 (Sacace Biotechnologie, como, Italie), selon les instructions du fabricant. Le programme d'amplification était le suivant: une dénaturation initiale de 15 minutes à 95° suivie de 42 cycles comprenant une dénaturation à 95°C pendant 15 secondes, une hybridation à 65°C pendant 25 secondes, une élongation à 72°C pendant 25 secondes et enfin une extension finale d'une minute à 72°C. Les fragments d'ADN amplifiés par PCR étaient séparés par électrophorèse sur un gel d'agarose à 2% (m/v) préparé dans une solution de tris base-borate-EDTA et contenant du bromure d’éthidium. Ces fragments étaient visualisés sous lumière UV. Le gel était ensuite photographié.

**Analyse statistique**: les données ont été analysées à l'aide des logiciels SPSS 17 .0 et Epi info 3.5.1. Le test de Chi carré a été utilisé pour les comparaisons. La différence a été significative pour p <0,05.

**Considérations éthiques**: le consentement libre et éclairé des sujets majeurs et celui des parents ou tuteurs légaux pour les mineurs a été obtenu avant la collecte. L’étude a été approuvée par le comité d’éthique institutionnel de Saint Camille-CERBA, Ouagadougou, Burkina Faso.

## Résultats

**Répartition des pathologies gastriques:** le Tableau 1 présente la répartition des pathologies gastriques en fonction des caractéristiques sociodémographiques des patients. Il ressort de ce tableau que les pathologies gastroduodénales n’étaient pas également reparties dans les différentes tranches d’âges. Les biopsies ont révélé respectivement 50% et 22,73% d'examens normaux chez les patients âgés de 16 à 25 ans et de 26 à 35 ans. Seuls 4,35% des patients âgés de plus de 45 ans présentaient un examen normal de biopsie. Les gastrites représentaient 23,64% chez les patients âgés de 16 à 35 ans et 61,74% chez ceux de plus de 36 ans. La fréquence des ulcères était plus élevée chez les patients âgés de plus de 45 ans (21,72%) comparativement aux patients âgés de 26 à 35 ans (13,64%). Aucun cas d'ulcère n'a été détecté dans la tranche d’âge de 16 à 25 ans. Un seul cas de cancer a été détecté chez une patiente de plus de 45 ans. De même un seul cas de lymphome à été détecté chez une patiente dans la tranche d’âge de 26 à 35ans. Les hernies représentaient 38,18% chez les patients de la tranche d’âge de 16 à 35ans et 13,04% chez les patients de plus de 45ans. Une fréquence plus élevée d'ulcère a été observée chez les hommes (23,33%) comparativement aux femmes (5,00%).

**Tableau 1:** Répartition des pathologies gastriques en fonction des caractéristiques sociodémographiques des patients

Pathologies gastriques		Examen Normal N (%)	Gastrite N (%)	Ulcère N (%)	Lymphome N (%)	Cancer N (%)	Hernie N (%)	Gastrite, +Ulcère+ Hernie, N (%)	Total N (%)
**Age**	16 - 25	5 (50,00)	1 (10,00)	0 (0,00)	0 (0,00)	0 (0,00)	2 (20,00)	2 (20,00)	10 (100)
	26 - 35	5 (22,73)	3 (13,64)	3 (13,64)	1 (4,54)	0 (0,00)	4 (18,18)	6 (27,27)	22 (100)
	36 - 45	3 (20,00)	6 (40,00)	1 (6,67)	0 (0,00)	0 (0,00)	3 (20,00)	2 (13,33)	15 (100)
	> 45	1 (4,35)	5 (21,74)	5 (21,74)	0 (0,00)	1 (4,35)	3 (13,04)	8 (34,78)	23 (100)
**Sexe**	Masculin	6 (20,00)	6 (20,00)	7 (23,33)	0 (0,00)	0 (0,00)	3 (10,00)	8 (26,67)	30 (100)
	Féminin	8 (20,00)	9 (22,50)	2 (5,00)	1 (2,50)	1 (2,50)	9 (22,50)	10 (25,00)	40 (100)

**Détection de H. pylori par PCR et test immunologique**: un fragment de 520 paires de base correspondant à H. pylori a été amplifié par PCR avec des échantillons de biopsies. Pour la totalité des 70 échantillons de biopsies gastriques analysées par la PCR, la prévalence de H. pylori a été de 91,43%, quant aux échantillons de sang analysés par le test ELISA Immunocomb, elle a été de 88,57% (Tableau 2). Ces résultats montrent une sensibilité et une spécificité de 92,2% et 50,0% pour la technique Immunocomb par rapport à la PCR.

**Tableau 2:** Comparaison des résultats de la PCR et de l'ELISA

Immunocomb	PCR		Total N(%)
	Positif N(%)	Négatif N(%)	
Positif N(%)	59 (84,28)	3 (4,28)	62 (88,57)
Négatif N(%)	5 (7,14)	3 (4,28)	8(11,43)
Total N(%)	64(91,43)	6(8,57)	70 (100)

Sensibilité = 92,2% (95% IC = 82; 97,1); Spécificité = 50,0% (95% IC =13,9; 86,1); V_pp_ = 95,2%; V_PN_ = 37,5%.

## Discussion

L'objectif principal de cette étude était l'usage de l'outil moléculaire (PCR) dans le diagnostic de H. pylori chez les patients venus pour une consultation de gastroentérologie au Centre Médical Saint Camille (Ouagadougou). Cette étude prospective portait sur 70 patients avec une prédominance féminine. La majorité des patients étaient des adultes dont l’âge était supérieur à 40 ans. Nous avons trouvé une prévalence élevée de H. pylori chez les patients en consultation de gastroentérologie au Centre Médical Saint Camille. La prévalence déterminée par le test PCR est nettement supérieure à celle obtenu par Ilboudo et al. (81,3%) au Burkina Faso et à celle identifiée par Fauchere en Côte d'Ivoire (69%) [5, 15]. Cependant la prévalence de H. pylori de cette étude est comparable aux résultats de Cataldo et al. (96,1%) [6]. De nos jours, la tendance à la transmission est à la hausse dans presque tous les pays en développement, ceci est dû aux conditions socioéconomiques des populations, à l'hygiène et à la promiscuité [16, 17]. Un autre problème augmentant la prévalence est le caractère asymptomatique de la bactérie dans le tube digestif pendant des années. En effet, nous avons identifié H. pylori chez 20% de patients ne présentant aucun problème gastrique par fibroscopie. Une stratégie de prévention contre l'infection préconise un diagnostic précoce de la bactérie. Dans de précédentes études, il a été montré que la prévalence de H. pylori augmentait avec l’âge [16, 18, 19]. Nos résultats montrent que les patients de la tranche d’âge de 16 à 25 ans et de 26 à 35 ans étaient les moins infectés par H. pylori et que les gastrites, les ulcères et les pathologies mixtes (gastrite, hernie, ulcère) étaient plus observés chez les patients les plus âgés. Ce qui est en accord avec les études antérieures. En effet, l'infection est contractée généralement très tôt dans l'enfance mais elle génère des complications pendant l’âge adulte, car la bactérie peut rester asymptomatique pendant des décennies.

Au Burkina Faso, le diagnostic de H. pylori se fait en routine essentiellement par les tests de dépistage rapide dont la sensibilité et la spécificité sont faibles. Cette technique de diagnostic présente l'inconvénient de ne pas pouvoir discriminer une infection active d'une infection guérie de H. pylori. Aussi, avons-nous utilisé la PCR comme méthode alternative de diagnostic chez les patients en consultation de gastroentérologie. En effet, la PCR a déjà été utilisée dans le diagnostic de H. pylori, dans d'autres études, et a montré une grande sensibilité (94%) et une spécificité élevée (100%) [20]. Dans la présente étude, nous avons également comparé la sensibilité et la spécificité de la technique sérologique Immunocomb par rapport à la PCR. La sensibilité et la spécificité du test Immunocomb étaient de 92,2% et 50,0%. Nous avons comparé la concordance des résultats de la PCR à ceux de l'Immunocomb: parmi les 70 patients diagnostiqués pour H. pylori, 64 étaient positifs à la PCR et 62 Positifs à l'Immunocomb. La concordance entre ces deux tests est de 84,28% (59/70) et une discordance de 11,42%. Ces résultats se rapprochent de ceux de Abdulqawik et al., qui avaient comparé une méthode de prélèvement invasif (Test Rapide à l'Urease) à une méthode sérologique (sérologie pour la recherche des anticorps IgG) et avaient trouvé une concordance de 88% et une discordance de 12% [21]. Il ressort donc que la PCR est plus sensible que la technique sérologique Immunocomb utilisée pour le diagnostic de H. pylori.

## Conclusion

Les résultats obtenus de cette étude suggèrent que le diagnostic de H. pylori par PCR est plus spécifique que le test sérologique Immunocomb et devrait être introduit, malgré son coût plus élevé, dans le diagnostic de routine de cette bactérie pathogène au Burkina Faso.
